# Digital technology adoption in dentistry: a comparative analysis of utilization, motivations, and barriers among dentists in Sibiu County, Romania

**DOI:** 10.25122/jml-2026-0099

**Published:** 2026-07

**Authors:** Andrei Andronic, Ionela Maniu, Maria Popa, Victoria Birlutiu

**Affiliations:** 1 Dental Medicine and Nursing Department, Faculty of Medicine, Lucian Blaga University, Sibiu, Romania; 2 Department of Mathematics and Informatics, Research Center in Informatics and Information Technology, Faculty of Sciences, Lucian Blaga University, Sibiu, Romania; 3 Pediatric Clinical Hospital Sibiu, Sibiu, Romania; 4 Faculty of Medicine, Lucian Blaga University, Sibiu, Romania; 5 Infectious Diseases Department, Academic Emergency Hospital Sibiu, Sibiu, Romania

**Keywords:** digital dentistry, digital technologies, technology adoption, adoption motivations, barriers to adoption, network analysis, dental practitioners, digital transformation, Romania, 3D, three-dimensional, CAD/CAM, computer-aided design/computer-aided manufacturing, COVID-19, coronavirus disease 2019, EBIC, extended Bayesian information criterion, GGM, Gaussian graphical model, gLASSO, graphical least absolute shrinkage and selection operator, M, motivation item, O, obstacle item, SD, standard deviation, TD, technology adoption item, TD1, patient scheduling and management software, TD2, practice/clinic management software, TD3, intraoral scanners, TD4, CAD/CAM systems, TD5, digital occlusal analysis tools, TD6, three-dimensional printers, M1, reduction of operational costs, M2, improvement of treatment quality, M3, improving the dentist–patient relationship, M4, increased clinical productivity, M5, keeping pace with technological advancements, M6, access to innovative materials and treatment techniques, M7, improved communication and workflow with the dental laboratory, M8, marketing and practice promotion purposes, M9, impact of the COVID-19 pandemic, O1, high cost of acquisition and implementation, O2, limited awareness of the advantages of digital technologies, O3, inadequate infrastructure, O4, insufficient theoretical and practical training, O5, lack of time for education and skill development, O6, difficulty adapting to and using digital technologies, O7, preference for conventional clinical methods

## Abstract

The rapid development of digital technologies has transformed dental practice by improving diagnostic accuracy, treatment quality, workflow efficiency, and patient management. However, the extent of technology adoption and the factors influencing its implementation vary considerably among dental professionals. This study aimed to evaluate the utilization of digital technologies among dentists in Sibiu County, Romania, compare adoption patterns between 2022 and 2025, and investigate the motivations and barriers associated with their implementation. We performed comparative statistical and network analyses to explore relationships among technologies, motivations, and obstacles. Patient scheduling and management software remained the most widely used digital technology in both survey periods, while intraoral scanners showed the greatest increase in adoption. Improvement of treatment quality, keeping pace with technological advancements, access to innovative materials and techniques, and increased productivity were identified as the main motivations for technology adoption. High acquisition and implementation costs remained the principal barrier in both surveys. User satisfaction was high across all technologies investigated, and most respondents expressed intentions to invest in digital technologies in the future. Network analysis revealed interrelationships among technologies, motivations, and barriers, and, in 2025 compared with 2022, indicated a maturation of the digitalization process. The adoption of digital technologies in dental practice increased between 2022 and 2025, reflecting the ongoing digital transformation of dentistry. Technology implementation was driven primarily by perceived clinical and professional benefits rather than by pandemic-related factors, while financial constraints remained the most important obstacle to wider adoption.

## Introduction

In recent decades, digitalization has become a major driver of change in healthcare systems, leading to medical services that are more efficient, accurate, and accessible. Today, the use of digital technologies in medicine is not just a technology upgrade. It is a strategy that directly affects the quality of healthcare delivery, the efficiency of clinical workflows, and the clinician-patient relationship [1,2]. In this changing environment, dentistry has been one of the medical specialties with the fastest and most dynamic digital transformation. This evolution has been enabled by the field’s highly technical, practice-oriented nature, as well as the increasing demand for personalized, predictable treatment strategies tailored to each patient’s clinical characteristics [3,4].

Current technological advancements involve integrating and applying an increasingly broad range of digital tools and systems, from intraoral scanners and computer-aided design/computer-aided manufacturing (CAD/CAM) systems to three-dimensional printing technologies and artificial intelligence in software designed for patient management and clinical practice administration [5,6]. Early digitalization in dentistry primarily focused on administrative tools. Patient and practice management software was progressively integrated into dental care settings, improving appointment scheduling, electronic record management, and internal workflow optimization [7,8]. These systems provided substantial logistical benefits and played a pivotal role in establishing a foundational digital infrastructure that facilitated the subsequent adoption and integration of advanced clinical technologies.

From a clinical perspective, intraoral scanners are among the most significant innovations in the digital transformation of dentistry. These devices provide a viable alternative to conventional impression techniques, offering greater efficiency, improved accuracy, and enhanced patient comfort during diagnostic and restorative procedures [9]. Revilla-León and colleagues reported that intraoral scanners are currently among the most widely adopted digital technologies in dental practice, owing to their reliability and seamless integration into existing clinical workflows [5].

Despite the high levels of satisfaction reported by clinicians who routinely use these systems, the literature continues to identify several barriers that may hinder their widespread adoption. Among the most frequently cited challenges are substantial initial investment costs, insufficient professional training opportunities, and difficulties integrating digital technologies into established clinical workflows [10–12].

Accordingly, findings reported by Hall MA and colleagues in Egypt indicate that although dental professionals widely acknowledge the benefits of digital technologies, the extent of their practical implementation varies considerably by demographic, educational, and economic factors [3]. Similarly, a study conducted by Radwan *et al*. in Saudi Arabia identified several challenges affecting the adoption of digital technologies, including financial constraints, insufficient training opportunities, and infrastructural limitations [4]. In the Netherlands, the adoption of digital technologies has been shown to be strongly influenced by the availability of appropriate clinical infrastructure and organizational support. Likewise, studies in the United Kingdom have shown that utilization of CAD/CAM systems remains lower than clinicians’ expressed willingness to implement them, primarily due to economic barriers and a lack of adequate professional training [9,10].

Integrating digital technologies into undergraduate dental curricula and continuing professional development programs has been associated with increased acceptance and use of these innovations among dental practitioners [12,13]. Furthermore, the adoption of CAD/CAM systems and other digital technologies appears to be higher among younger professionals and dental students who have received structured digital training during their academic education [9]. In this context, professional education and training emerge as strategic determinants in accelerating the digital transformation of dental practice and promoting the sustainable implementation of digital technologies in routine clinical care.

The adoption of digital technologies in dentistry is driven by a range of clinical and organizational factors, including the potential to enhance diagnostic accuracy, improve treatment outcomes, increase clinical efficiency, streamline workflows, and integrate technological innovation into everyday dental practice. Collectively, these advantages are key drivers of digital transformation, supporting the delivery of more precise, predictable, and patient-centered dental care [14–16].

The digital transformation of dentistry is a complex, multifaceted process that requires a comprehensive understanding of the factors influencing technology adoption, the mechanisms by which existing barriers can be overcome, and the strategies needed to maximize the clinical and organizational benefits of emerging digital technologies.

The rapid advancement of digitalization in dentistry, together with the continuous expansion of digital tools available for clinical practice, highlights the need for an in-depth evaluation of how these technologies are adopted and integrated into dentists’ professional activities in Romania. As intraoral scanning, practice management software, CAD/CAM systems, three-dimensional printing, and digital occlusal analysis technologies play an increasingly important role in workflow optimization, diagnostic accuracy, and treatment quality, it is essential to investigate their actual level of use and the factors that facilitate or hinder their adoption.

The present research was motivated by the need to better understand the dynamics of digitalization among dentists practicing in Sibiu County, a professional community undergoing an active technological transition while also facing challenges related to implementation costs, infrastructure availability, professional training, and perceptions of the usefulness of digital technologies. By exploring these aspects, the study seeks to provide a current and comprehensive overview of how digital technologies are perceived, implemented, and utilized in local dental practice, thereby generating evidence that may support the development of effective strategies for modernization and adaptation to the evolving demands of contemporary healthcare.

The primary objective of this study was to assess the extent of digital technology utilization among dentists in Sibiu County, analyze changes in adoption patterns between the two evaluation periods (2022 and 2025), and evaluate practitioners’ satisfaction with integrating these technologies into routine clinical practice. In addition, the study aimed to investigate the motivations underlying the acquisition and use of digital technologies, including the pursuit of improved quality of care, alignment with technological advancements, workflow optimization, access to innovative materials and treatment techniques, and enhancement of the dentist–patient relationship. Furthermore, the study sought to identify the principal barriers limiting the widespread adoption of digital technologies in dental practice. Collectively, these objectives were designed to provide a comprehensive perspective on the current state of digitalization in dental clinics within Sibiu County, Romania, and to contribute to a better understanding of the factors shaping its future development.

## Material and Methods

A cross-sectional study was conducted to achieve the research objectives and collect the data required for the analysis. Data were collected using a non-probability convenience sampling approach through a structured online questionnaire developed and distributed via the Google Forms platform. Invitations to participate were sent by email to all dentists practicing in Sibiu County, Romania.

The questionnaire was developed after reviewing the relevant scientific literature and was adapted to the objectives and scope of the study. A panel of medical experts and a statistician affiliated with Lucian Blaga University of Sibiu evaluated the initial version of the instrument and provided recommendations on the clarity, relevance, and consistency of the questionnaire items.

The final questionnaire was organized into two sections. The first section included six items designed to collect participants’ sociodemographic and professional characteristics, including age, sex, years of professional experience, and type of dental practice. The second section comprised items assessing the utilization of digital technologies in dental practice, the level of satisfaction associated with their use, and the motivations and barriers related to their adoption.

Before distribution, the questionnaire was pilot-tested among five practicing dentists to evaluate the clarity, comprehensibility, and relevance of the items. The final version of the questionnaire was distributed electronically to the entire dental professional community in Sibiu County. The Sibiu County College of Dentists facilitated the dissemination of the survey invitation to its affiliated members. The data collection period lasted 3 months for the 2022 survey (April–June 2022) and 2 months for the 2025 survey (July–August 2025). During both data collection periods, two reminder messages were distributed through WhatsApp to encourage participation.

Participation was voluntary. Prior to completing the questionnaire, all participants provided informed consent and were assured of the anonymity and confidentiality of the collected data. A total of 131 responses were obtained from 600 eligible dentists in 2022, corresponding to a response rate of 21.8%, while 198 responses were collected from 630 eligible dentists in 2025, corresponding to a response rate of 31.4%. The participation rate increased by 9.6 percentage points between the two survey periods.

The variables were reported as frequency and percentage or as mean and standard deviation (SD). Parametric and nonparametric tests were used to compare the two evaluation periods: the chi-square test for professional qualification/experience, age, sex, practice location, and number of dentists employed in the practice, and the Student *t*-test for motivations and barriers. All statistical tests were two-tailed. *P* values are presented without adjustment for multiple comparisons. We analyzed the interrelationships between technology adoption items (TD), perceived obstacles items (O), and internal motives items (M) using a network analysis approach. In this network, nodes represent the variables (nodes that are more strongly connected are pulled closer together), and edges represent partial correlation coefficients after controlling for all other variables. Network architecture was estimated using a Gaussian graphical model (GGM) with the graphical lasso (gLASSO) regularization technique combined with the extended Bayesian information criterion (EBIC) for the selection of the optimal tuning parameter, the walktrap algorithm for the determination of the number of dimensions, and the Fruchterman-Reingold algorithm for the spatial arrangement of the nodes (where node proximity reflects partial correlation strength; green edges indicate positive associations, red edges indicate negative associations, and edge thickness represents association strength). We calculated partial correlations based on polychoric correlations, and set the EBIC hyperparameter to 0.5. The data were analyzed using R version 4.0.5 (R Foundation for Statistical Computing, Vienna, Austria).

## Results

### Demographic and professional characteristics of the participants

The age distribution of respondents in the 2025 survey showed that the largest proportion of participants were aged over 40 years (52.02%), followed by those aged 31–40 years (33.33%). By comparison, the 2022 survey showed a different age distribution. The largest proportion of respondents were aged 31–40 years (47.33%), followed by those aged over 40 years (29.01%). Regarding sex distribution, female dentists constituted the majority of respondents in both survey periods (2022: 61.07% and 2025: 62.12%). Regarding professional qualification, 67.94% of participants in 2022 and 66.67% in 2025 identified as general dental practitioners. Analysis of professional experience demonstrated comparable proportions of respondents with 11–20 years of clinical practice, accounting for 41.22% of participants in 2022 and 40.91% in 2025. Furthermore, 14.50% of respondents in 2022 and 25.76% in 2025 reported more than 20 years of professional experience. Regarding workplace characteristics, most respondents reported practicing in small dental offices employing one or two dentists (2022: 45.80% and 2025: 52.02%). Medium-sized practices, employing three to five dentists, represented the workplace setting for 31.30% of respondents in 2022 and 24.75% in 2025. In 2022, 22.90% of respondents and 23.23% in 2025 were from large dental clinics with more than five dentists. Most participants in both survey periods practiced in urban areas. These characteristics are presented in Table 1.

**Table 1:** Characteristics of participants

Characteristics	2022	2025	*P* value
Professional qualification			0.847
Specialist	42 (32.06%)	66 (33.33%)	
Generalist	89 (67.94%)	132 (66.67%)	
Age			0.000
20–30 years	31 (23.66%)	29 (14.65%)	
31–40 years	62 (47.33%)	66 (33.33%)	
>40 years	38 (29.01%)	103 (52.02%)	
Sex			0.848
Male	51 (38.93%)	75(37.88%)	
Female	80 (61.07%)	123(62.12%)	
Professional experience in dentistry (years)			0.053
<5 years	30 (22.90%)	30 (15.15%)	
5-10 years	28 (21.37%)	36 (18.18%)	
11-20 years	54 (41.22%)	81 (40.91%)	
>20 years	19 (14.50%)	51 (25.76%)	
Practice location			0.118
Urban	127 (96.95%)	184 (92.93%)	
Rural	4 (3.05%)	14 (7.07%)	
Number of dentists employed in the practice			0.395
1–2	60 (45.80%)	103 (52.02%)	
3–5	41 (31.30%)	49 (24.75%)	
>5	30 (22.90%)	46 (23.23%)	

### Utilization of digital technologies

**Figure 1:**
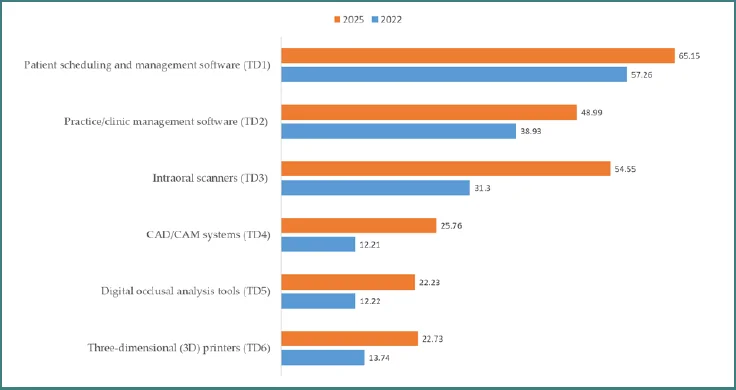
Utilization of digital technologies in 2022 and 2025. CAD/CAM, computer-aided design/computer-aided manufacturing; TD1, patient scheduling and management software; TD2, practice/clinic management software; TD3, intraoral scanners; TD4, CAD/CAM systems; TD5, digital occlusal analysis tools; TD6, three-dimensional printers.

Among the digital technologies evaluated, patient scheduling and management software (TD1) showed the highest adoption, reported as routinely used (2022: 57.26% and 2025: 65.15%). This finding highlights its role as an essential tool in daily dental practice. In 2022, intraoral scanners (TD3) ranked third in terms of utilization (31.30%), following patient management software (TD1) and practice/clinic management software (TD2) (38.93%). By 2025, however, intraoral scanners had risen to second place, with 54.55% of respondents reporting their use, surpassing practice management software (48.99%) (Figure 1).

Conversely, the technologies with the lowest levels of integration into routine dental practice were CAD/CAM systems (TD4) (2022: 12.21% and 2025: 25.76%), digital occlusal analysis tools (TD5) (2022: 12.22% and 2025: 22.23%), and three-dimensional (3D) printers (TD6) (2022: 13.74% and 2025: 22.73%) (Figure 1). Although all three technologies exhibited increased adoption over time, their overall utilization rates remained relatively low. Despite their limited current use, these technologies generated the greatest interest among respondents who had not yet adopted them. The proportion of participants reporting that they were not currently using, but intended to implement, digital occlusal analysis tools reached 64.89% in 2022 and 64.14% in 2025. Similarly, 61.07% of respondents reported interest in adopting 3D printing technology in 2022 and 56.06% in 2025, while CAD/CAM systems attracted interest from 58.02% and 51.52% of respondents, respectively. A substantial level of interest was also observed for intraoral scanners, although the proportion of respondents expressing future adoption intentions decreased from 54.96% in 2022 to 38.38% in 2025, likely reflecting their increased implementation during the study period. Interest in practice management software remained relatively stable (2022: 39.69% and 2025: 36.87%). Lower levels of future adoption interest were reported for patient management software (2022: 29.77% and 2025: 26.77%). In contrast, a notable proportion of respondents reported neither using nor intending to adopt certain technologies. Nevertheless, this proportion declined between the two survey periods. For CAD/CAM systems, the percentage of respondents expressing no interest decreased from 29.77% in 2022 to 22.73% in 2025, while for 3D printers it declined from 25.19% to 21.21%, suggesting a gradual increase in the overall acceptance of advanced digital technologies within the dental profession.

### Satisfaction with the use of digital technologies

Satisfaction among users of digital technologies was high, as reflected in their willingness to recommend these solutions to professional colleagues. Among dentists using patient scheduling and management software, 93.3% of respondents in the 2022 survey and 93.7% in the 2025 survey reported that they would recommend this technology to other practitioners. Similarly, a high level of satisfaction was observed among users of intraoral scanners. The proportion of respondents willing to recommend this technology increased from 82.3% in 2022 to 92.5% in 2025, indicating a growing appreciation of its clinical benefits. High recommendation rates were also reported for the other digital technologies evaluated. Among users of practice or clinic management software, 82.3% in 2022 and 85.5% in 2025 stated that they would recommend these systems to colleagues. For digital occlusal analysis tools and software, the proportion of respondents willing to recommend their use increased from 68.7% in 2022 to 84.09% in 2025. Likewise, recommendation rates for three-dimensional (3D) printing technologies rose from 66.6% in 2022 to 82.2% in 2025. 72.5% of CAD/CAM users reported that they would recommend these systems to other dentists, a slight decline from 75.0% in 2022.

### Motivations for the adoption of digital technologies

Most respondents considered improved treatment quality (M2) the primary motivation for adopting digital technologies in dental practice (2022: 3.96 ± 1.22; 2025: 4.12 ± 1.26). Another highly rated motivation was the desire to keep pace with technological advancements (M5), which received mean scores of 3.86 in 2022 and 3.98 in 2025. Similarly, access to new materials and treatment techniques (M6) was identified as an important driver of adoption (2022: 3.83 ± 1.07; 2025: 3.90 ± 1.32). Respondents also emphasized the role of digital technologies in increasing clinical productivity (M4) and improving the dentist–patient relationship (M3) (Table 2). Furthermore, dentists perceived digital technologies as beneficial for enhancing communication and workflow efficiency with dental laboratories (M7, 2022: 3.56 ± 1.28; 2025: 3.67 ± 1.43). These findings suggest that improvements in interdisciplinary collaboration provide an additional incentive for adopting digital solutions. In contrast, using digital technologies as a marketing tool (M8), their potential to reduce operational costs (M1), and adoption in response to circumstances generated by the COVID-19 pandemic (M9) were considered less important motivations (Table 2).

**Table 2:** Motivations for the adoption of digital technologies

Motivation	2022	2025	*P* value
Reduction of operational costs (M1)	2.27 ± 1.26	2.55 ± 1.39	0.091
Improvement of treatment quality (M2)	3.96 ± 1.22	4.12 ± 1.26	0.068
Improving the dentist–patient relationship (M3)	3.55 ± 1.25	3.78 ± 1.39	0.035
Increased clinical productivity (M4)	3.67 ± 1.30	3.81 ± 1.35	0.200
Keeping pace with technological advancements (M5)	3.86 ± 1.18	3.98 ± 1.29	0.110
Access to innovative materials and treatment techniques (M6)	3.83 ± 1.07	3.90 ± 1.32	0.130
Improved communication and workflow with the dental laboratory (M7)	3.56 ± 1.28	3.67 ± 1.43	0.200
Marketing and practice promotion purposes (M8)	2.89 ± 1.38	2.93 ± 1.56	0.800
Impact of the COVID-19 pandemic (M9)	2.35 ± 1.31	2.11 ± 1.34	0.053

### Barriers to the adoption of digital technologies

High costs of acquisition and implementation (O1), including expenses related to equipment procurement and software licensing, were perceived as the most significant barrier to the adoption and implementation of digital technologies in dental practice (2022: 4.03 ± 1.12; 2025: 3.76 ± 1.33), indicating that financial considerations remain the principal obstacle to digital transformation among dentists. Other barriers were generally perceived as having a moderate influence on technology adoption. These included limited awareness of the benefits associated with digital technologies (O2; 2022: 2.74 ± 1.25; 2025: 2.75 ± 1.30), inadequate infrastructure (O3; 2022: 2.92 ± 1.27; 2025: 2.60 ± 1.27), insufficient theoretical knowledge and practical skills (O4; 2022: 2.73 ± 1.25; 2025: 2.62 ± 1.28), and lack of time available for training and professional development (O5; 2022: 2.62 ± 1.27; 2025: 2.52 ± 1.27). Of these, only inadequate infrastructure differed significantly between the two survey periods (*P* = 0.019). By contrast, difficulties related to the general use of technology (O6) and practitioners’ preference for conventional clinical methods (O7) were regarded as relatively minor obstacles (Table 3).

**Table 3:** Barriers to the adoption of digital technologies

Barriers	2022	2025	*P* value
High cost of acquisition and implementation (O1)	4.03 ± 1.12	3.76 ± 1.33	0.122
Limited awareness of the advantages of digital technologies (O2)	2.74 ± 1.25	2.75 ± 1.30	0.923
Inadequate infrastructure (O3)	2.92 ± 1.27	2.60 ± 1.27	0.019
Insufficient theoretical and practical training (O4)	2.73 ± 1.25	2.62 ± 1.28	0.400
Lack of time for education and skill development (O5)	2.62 ± 1.27	2.52 ± 1.27	0.500
Difficulty adapting to and using digital technologies (O6)	2.12 ± 1.23	2.11 ± 1.24	0.957
Preference for conventional clinical methods (O7)	2.14 ± 1.31	2.09 ± 1.29	0.700

### Network analysis of digital technologies (TD), adoption motivations (M), and perceived barriers (O)

**Figure 2:**
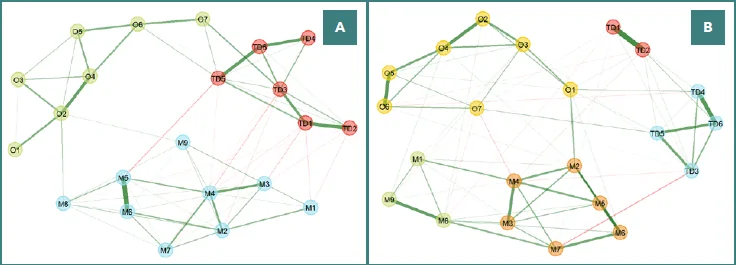
Network structure of the interconnections between technologies (TD), obstacles (O), and motives (M) in (A) 2022 and (B) 2025. Green lines represent positive partial correlations and red lines represent negative partial correlations between nodes; edge thickness represents the strength of the association. TD1, patient scheduling and management software; TD2, practice/clinic management software; TD3, intraoral scanners; TD4, computer-aided design/computer-aided manufacturing (CAD/CAM) systems; TD5, digital occlusal analysis tools; TD6, three-dimensional (3D) printers; M1, reduction of operational costs; M2, improvement of treatment quality; M3, improving the dentist–patient relationship; M4, increased clinical productivity; M5, keeping pace with technological advancements; M6, access to innovative materials and treatment techniques; M7, improved communication and workflow with the dental laboratory; M8, marketing and practice promotion purposes; M9, impact of the COVID-19 pandemic; O1, high cost of acquisition and implementation; O2, limited awareness of the advantages of digital technologies; O3, inadequate infrastructure; O4, insufficient theoretical and practical training; O5, lack of time for education and skill development; O6, difficulty adapting to and using digital technologies; O7, preference for conventional clinical methods.

Network analysis explored the structural relationships among digital technologies (TD), adoption motivations (M), and perceived barriers (O) (Figure 2). For nodes related to the adoption of digital technologies (TD), a temporal reconfiguration was observed between 2022 and 2025. In 2022, intraoral scanning (TD3) exhibited an intermediate position, acting as a bridging node between administrative and clinically oriented technologies, consistent with a transitional role in the digitalization process. In 2025, TD3 showed stronger edge weights within the clinical technology cluster (TD3–TD6), indicating integration into a more cohesive clinical digital ecosystem and a shift toward higher functional embedding in routine workflows.

For nodes representing motives (M), the network showed the highest density and structural cohesion across both time points, with consistently strong intra-cluster connectivity. Representative nodes included improvement of treatment quality (M2), increased productivity (M4), technological advancement (M5), and adoption of new materials and techniques (M6), which jointly formed the dominant motivational core. In 2022, pandemic-related motivation (M9) showed increased betweenness centrality, reflecting a context-dependent driver of adoption. By 2025, M2 emerged as the dominant centrality node, indicating consolidation of clinically oriented motivational pathways and reduced external contextual influences.

The obstacle (O) nodes exhibited marked structural evolution over time. In 2022, the network showed lower density and a more dispersed topology, with limited clustering and a central substructure composed primarily of O2, O4, and O5. Cost-related (O1) and infrastructure-related (O3) barriers demonstrated peripheral positioning with lower connectivity strength. In 2025, the barrier network became more compact and highly interconnected, with increased global density and a clearly defined central cluster including O2, O3, O4, O5, and O6. This shift indicates greater structural integration and stronger interdependencies among perceived constraints.

Cross-network analysis revealed inter-cluster edges. In 2022, O2 showed cross-domain connectivity with M8 and M9, suggesting links between perceived informational barriers, marketing-oriented motivations, and contextual pandemic effects. Additionally, O7 demonstrated weak negative associations with TD3 and TD5, indicating an inverse relationship between preference for conventional methods and adoption of advanced digital technologies. In 2025, cross-network connectivity persisted with modified structural patterns. O1 exhibited associations with M2, suggesting that perceived financial barriers coexist with strong clinical value attribution. Furthermore, O7 maintained connections with M4 and M8, indicating that resistance to technological change remains partially embedded within productivity and strategic positioning motivations. Overall, the network evolution suggests increasing structural integration of barriers, motivations, and technologies, alongside a gradual strengthening of clinically driven centrality patterns in the digital transformation of dental practice.

## Discussion

The comparative analysis of the two studies conducted in different years (2022 and 2025) highlights a relatively stable respondent profile, with several characteristics that have remained unchanged or have become more pronounced over time. In 2022, the predominant age group was 31–40 years (47.33%); in 2025, the distribution shifted toward dentists over 40 years of age (52.02%), which may suggest a greater involvement of more experienced practitioners in specialized studies. Regarding sex distribution, the structure remained almost unchanged, with a consistent female majority of approximately 62% in both studies. In terms of professional training, the proportions remained similar, with approximately two-thirds of respondents being general dental practitioners and one-third specialists. The level of professional experience shows an upward trend: while in 2022, 55.72% of participants reported more than 11 years of practice, in 2025 this proportion increased to 66.67%. The working environment remains dominated by small dental practices employing one or two dentists, with their proportion increasing from 45.8% in 2022 to 52.02% in 2025. At the same time, the geographical distribution remains predominantly urban, although it decreases slightly, from 96.95% in 2022 to 92.93% in 2025.

The demographic and professional characteristics of the participants describe a mature, professionally experienced sample whose decisions about adopting and using digital technologies are likely influenced by both clinical and economic considerations, including quality, workflow integration, and cost–benefit ratio. The prevalence of small dental practices and the concentration of professional activity in urban areas justify a steady adoption of easily integrated technologies, alongside a cautious attitude toward major investments associated with higher risk. The obtained data are consistent with the international literature regarding the predominance of the female gender [17,18], the predominance of small private practices and the higher proportion of general dental practitioners [19,20], as well as the uneven urban–rural distribution of oral health services, which are consistent with findings reported in the international literature [19,21].

The analysis of the utilization level of various digital technologies in dental practice reveals a clear distinction between basic or administrative tools and advanced clinical technologies. A first relevant finding is the consolidation of foundational digital solutions, particularly patient management applications. These remain the most frequently used digital tools (57.26% in 2022 and 65.15% in 2025), confirming their essential role in organizing and optimizing clinical workflows. Patient record systems and appointment management software have become nearly ubiquitous, used by approximately two-thirds of dentists, while most non-users express interest in adopting them. This trend highlights that the digitalization of administrative processes—from electronic patient records and online scheduling to database management—is perceived as a natural step, relatively easy to implement, and associated with manageable costs. Furthermore, in many countries, electronic health records have become a de facto standard because they improve operational efficiency, support compliance with data protection regulations and mandatory reporting requirements, and facilitate interdisciplinary communication [13]. Similar results have been reported in Canada, where these solutions have become the core digital infrastructure of dental practices [22]. In Saudi Arabia, Radwan *et al*. emphasized their major role in improving administrative efficiency and patient retention [4]. Higher adoption rates have also been documented in Germany [13] and the Netherlands [10]. Differences in adoption rates may be explained by local economic factors, as well as by the gradual nature of the digitalization process within the Romanian healthcare system. Between 2022 and 2025, the use of intraoral scanners increased significantly, rising from 31.34% to 54.55%. This technology moved to second place in the hierarchy of currently used digital technologies, surpassing clinic management software. While intraoral scanning was considered a rare practice a decade ago, more than half of the dentists in the sample now use it, reflecting recognition of its clinical advantages: highly accurate impressions, increased patient comfort, and reduced working time [6,13].

These findings align with those of Revilla-León *et al*., who regard intraoral scanning as the contemporary standard for digital impression acquisition in modern dentistry [5]. In India [23], the utilization rate is 49%, and in Germany [13], 51%, values comparable to those reported in Romania. In contrast, higher levels of integration have been reported in the United States (53% in 2021 [5]) and China (72,17% [24]). International studies [25,26] indicate increasing daily use and a progressive acceleration of intraoral scanning integration into clinical workflows, a trend also reflected in the pattern observed among dentists from Sibiu. Consequently, this technology is no longer perceived solely as an innovation but has become an integrated tool in routine clinical activity and dental practice in Romania. The adoption rate is expected to continue to increase, driven by the emergence of more cost-accessible scanner models and the increasingly widespread digital processing of impressions by dental technicians, which will further support the transition toward a fully digital workflow in dental practice. Moreover, intraoral scanning technology shows not only changes in its level of utilization but also in its positioning within the overall technology network. In the 2022 network structure, intraoral scanning (TD3) occupied a central, intermediary position between administrative and clinical technologies, suggesting its role as a transitional technology in the digitalization process. By 2025, the same technology became fully integrated within the cluster of clinical digital technologies, establishing strong connections with CAD/CAM systems, 3D printing, and digital occlusal analysis tools. This shift indicates that intraoral scanners no longer function as a transitional technology between different stages of digital adoption, but rather as a fully embedded component of the digital clinical workflow. In contrast to basic digital technologies, advanced digital tools such as CAD/CAM systems, 3D printing, and occlusal analysis software continue to be less frequently used, although they recorded moderate increases in adoption rates between 2022 and 2025 (CAD/CAM: 12.21% → 25.76%; 3D printing: 13.74% → 22.73%).

At the international level, adoption rates vary significantly. In China, CAD/CAM utilization is reported at 42.45% [24], in Saudi Arabia at 41.2% [27], and in Germany at 48% [13]. Regarding 3D printing, implementation in Romania exceeds that reported in the United States (17%) [5] but remains lower than in Germany (32%) [13] or China (37,74%) [24]. The low level of adoption can be explained by the high costs of acquiring and using these technologies, the need for additional investments in infrastructure and professional training, and the difficulties of integrating them into routine clinical workflows. These findings are consistent with the study conducted by Tran *et al*. in the United Kingdom, which highlighted that CAD/CAM adoption is hindered by financial barriers and challenges related to integration into daily practice [9]. Therefore, economic and organizational factors appear to continue to limit the widespread adoption of advanced digital technologies in dentistry, despite their well-documented clinical benefits. Digital occlusal analysis tools recorded the lowest utilization rate (22.23%). Systems such as T-Scan and similar technologies remain niche instruments, as they are not perceived as essential in routine dental practice. Their utility is primarily acknowledged in complex oral rehabilitation cases and among practitioners with a strong interest in occlusion. However, 64% of dentists who do not currently use these systems expressed an intention to adopt them in the future. This high level of interest suggests that non-users also recognize their clinical benefits, which may contribute to broader adoption in the future, particularly as these technologies become more financially accessible [28].

A similar trend is observed with 3D printing and CAD/CAM systems, where more than half of dentists who do not currently use these technologies (56.06% for 3D printing and 51.52% for CAD/CAM) reported an intention to adopt them in the future. The interest expressed by non-users suggests favorable conditions for increased future use of these technologies.

The discrepancy between current usage levels and expressed interest indicates that the adoption of these technologies is limited primarily by objective barriers, such as high equipment costs and the need for additional training for integration into clinical practice, rather than by a lack of professional interest or insufficient awareness of their benefits [2,4].

For already established core technologies, such as patient management software and intraoral scanning, declared interest in future adoption is lower and even shows a slight decline. This decrease in interest does not reflect reduced attractiveness; it reflects increased utilization, as more clinicians have moved from adoption intention to full integration of these technologies into routine practice. The high level of interest expressed by dentists from Sibiu County regarding the future adoption of digital technologies is consistent with findings reported in other international studies. In the United States, the use of 3D printing in dental practices remains relatively limited (approximately 17%), yet the intention to invest in and engage in professional training among non-users is evident: 21% of respondents intend to acquire such systems, and 35% report willingness to participate in training programs [29]. Similarly, for intraoral scanning, a survey conducted by ACE/JADA showed that although 53% of respondents already use this technology, 34% of non-users are considering acquiring an intraoral scanner, while 40% intend to participate in training courses [5].

Between 2022 and 2025, dentists’ willingness to recommend digital technologies remained high, with a slight upward trend for most technologies. This evolution suggests that once the initial implementation and active-use threshold is reached, dentists tend to become promoters of the technology, showing increased willingness to recommend it to both colleagues and patients. The obtained data are comparable with international findings, where 91% of intraoral scanner users report a high level of satisfaction [5]. In contrast, for 3D printing, although the adoption rate remains modest, the perceived advantages, particularly those related to efficiency and cost reduction, are clearly recognized. This combination of user satisfaction and perceived benefits explains users’ increased willingness to recommend these technologies [29]. In contrast, satisfaction with CAD/CAM systems declines from 78.9% to 72.5%, placing them last in the 2025 ranking. However, international data indicate a significant difference: a study conducted by Krastev *et al*. in Austria reported a recommendation rate of 96.2% [30]. The lower level of satisfaction observed at the local level may be explained by the technological complexity of these systems, the high acquisition and maintenance costs, as well as the steep learning curve, factors that limit their optimal integration into routine clinical practice [31].

The motivation for adopting digital technologies in dentistry is primarily driven by concerns related to improving the quality of clinical care and modernizing professional practice. “Improvement of treatment quality” (M2) represents the most frequently cited adoption motive (3.96 → 4.12), followed by the “need to keep pace with technological progress” (M5) (3.86 → 3.98). Both factors were the main drivers of adoption in 2022, and the 2025 results confirm their persistence and further consolidation, suggesting that investments in digital technologies are driven primarily by perceived clinical value and the ongoing need for modernization. In the study by Alyami *et al*., more than half of participants (58.1%) stated that digital technologies improve treatment quality. Similarly, clinical efficiency was reported as the main reason for acquiring intraoral scanners by 70% of participants in the American Dental Association (ADA) study [5,27]. Additionally, 40% of respondents considered this technology to provide superior outcomes compared to conventional methods [5], confirming the trend also observed among participants from Sibiu.

A similar trend was observed between 2022 and 2025 for other motivations associated with digitalization, such as the use of new materials and techniques (M6), increased productivity (M4), and improved dentist–patient relationships (M3). Respondents also maintained a positive perception of improved communication and workflow with the dental laboratory (M7). These changes suggest a more professional perspective on digitalization, in which clinical and organizational benefits are perceived as complementary.

In contrast, digital technologies are not perceived as strong enough marketing tools to justify the effort of implementation (M8), while economic motivations such as cost reduction (M1) remain secondary in decisions about adopting digital technologies. Similarly, the pandemic context (M9) is the only motivation that decreases in relevance, suggesting a predominantly circumstantial role. Network analysis complements and supports the conclusions derived from the descriptive analysis. In 2022, modernization and digitalization of the dental practice (M5) appear closely associated with the use of innovative materials and techniques (M6), as well as improvements in treatment quality (M2) and practice productivity (M4). Improvement of treatment quality already occupied a central position in the motivational structure in 2022, being associated with patient satisfaction (M3) and increased clinical efficiency (M4).

In 2025, network analysis reveals the motivational domain organized into two subclusters. The first, represented by variables M2–M7, reflects a predominantly professional and clinical motivational model centered on modernizing clinical practice, optimizing workflow, and enhancing treatment quality. The increased network density and the central positioning of M2 indicate that respondents perceive digitalization as an integrated process, in which technological advancement, new materials and techniques, clinical efficiency, and patient satisfaction simultaneously contribute to improved treatment quality.

In contrast, the second subcluster, formed by M1, M8, and M9, reflects a secondary set of motivations that are less integrated into the clinical dimension of digitalization. Its peripheral positioning suggests that cost reduction, practice marketing, and pandemic-related factors do not represent primary determinants of technology adoption, but rather complementary benefits or contextual drivers.

Although nearly three-quarters of the surveyed dentists (73.2%) believe digital technologies improve the quality of dental treatments, implementation remains low. The main barriers identified in our study, namely equipment cost, recurring expenses, the need for training, and the learning curve, are also reported in the international literature [1,32].

High costs (O1) associated with acquiring equipment, software licenses, and data processing programs, along with subsequent maintenance expenses, are perceived as the major obstacle to the widespread adoption of digital technologies. Although the perceived financial barrier shows a slight decrease, it remains the most significant impediment reported by participants in our study. In a study conducted in Saudi Arabia [27] and published in 2025, 44.5% of participants indicated cost as the main barrier to implementing digital technologies. Canadian dentists (63%) [22] reported the same primary barrier, as did their counterparts in India (37%) [2]. Regarding the acquisition of 3D printers, 39% of dentists participating in an American Dental Association study reported being discouraged by high costs [33]. Regardless of geographical context or healthcare system characteristics, dentists perceive acquisition and maintenance costs as major obstacles. The concordance between international data and the findings of the present study suggests that financial barriers are not only a local issue but a global impediment that limits the pace and extent of digitalization in dentistry.

Lack of infrastructure (O3), insufficient knowledge regarding the advantages of these technologies (O2), limited theoretical and practical training (O4), and lack of time for training (O5) fall within the category of moderate barriers. The negative perception associated with these factors has slightly decreased between 2022 and 2025, which may suggest a gradual adaptation of dentists to current digitalization trends.

The network analysis of barriers in 2022 highlights the relationship between insufficient information regarding the advantages of digital technologies (O2), limited theoretical and practical knowledge required for their use (O4), and lack of time for training (O5). The connection between these variables, together with their association with high costs (O1), indicates an economic–educational barrier that can limit the adoption of digital technologies. Implementation difficulties are not exclusively financial but also involve access to information, professional training, and the availability of educational resources. In this context, the need for dedicated professional training programs in digital dentistry becomes evident, especially since undergraduate curricula have not yet sufficiently integrated these relatively recent areas of development.

In 2025, the more compact and interconnected structure of the barrier network suggests consolidating and integrating the economic–educational barrier into a more complex model of perceived difficulties associated with digitalization. The formation of a central core consisting of O2, O3, O4, O5, and O6 indicates a close relationship between information level, professional training, available infrastructure, and practical capacity to use digital technologies.

The persistent strong link between O2 and O4 further supports the relevance of continuing professional education and training programs dedicated to the digitalization process. At the same time, the relationship between O5 and O6 suggests that insufficient time allocated to training may directly contribute to difficulties in using digital technologies, indicating that practical and educational barriers tend to reinforce each other. These trends align with international studies on digitalization in healthcare, which show that inadequate infrastructure, lack of competencies, and high workload are frequent barriers. Conversely, dedicated training programs and a clear perception of the usefulness of technologies have been identified as key factors in facilitating the adoption process [32].

General difficulties in using technology (O6) or preference for conventional methods (O7) were assessed as minor barriers, and perceptions of these factors remained virtually unchanged over the three-year period. The association between O7 and O6, in both 2022 and 2025, may indicate a distinct subgroup of respondents characterized by general difficulties adapting to technology and a preference for conventional working methods. These obstacles appear to reflect predominantly behavioral factors rather than structural limitations of the digitalization process. Only a small proportion of the sample considered resistance to change from the clinician or the dental team, or habituation to traditional methods, as real barriers to implementing digital technologies. This finding suggests openness among participating dentists toward innovation and a willingness to accept change when adequate conditions are in place, such as appropriate information, training programs, and financial support. Similar results have been reported in other studies, where healthcare professionals’ resistance was shown to be a secondary factor compared with practical barriers, particularly costs and the need for professional training [2,13].

Interconnections between variables in different groups provide additional information on the motivations and factors influencing the adoption of digital technologies, as well as how these factors interact.

Thus, in 2022, the relationship between O2 (insufficient information regarding the advantages of these technologies) and M8 (use as a marketing tool), as well as M9 (pandemic context), may suggest that limited awareness of the benefits of digital technologies reduces the perceived role of these tools in practice promotion and in adapting dental activity to the COVID-19 pandemic context. The association between O7 (preference for conventional methods) and the level of adoption of TD3 (intraoral scanning/digital impression) and TD5 (digital tools/software for occlusal analysis) may indicate that technologies directly modifying traditional clinical workflows and techniques encounter a higher degree of reluctance among practitioners with a more conservative professional orientation. The link between TD5 (occlusal analysis tools/software) and M5 (desire to keep pace with technological progress) may suggest that use of these technologies is driven less by modernization motivation or innovation orientation and more by specific clinical needs. The association between the use of TD1 (patient management software) and M3 (improvement of the dentist–patient relationship) may indicate that respondents do not perceive administrative software as having a direct impact on the dentist–patient relationship, considering it primarily an administrative organization and efficiency tool. The links between M7 (improved communication and workflow with the dental laboratory) and TD3 (intraoral scanning), as well as TD4 (CAD/CAM systems), may suggest that these technologies partially replace conventional stages of collaboration with the dental laboratory, being perceived predominantly as tools for internal digitalization of the dental practice.

In 2025, the relationship between O1 (high cost) and M2 (improvement of treatment quality) may suggest that respondents who recognize the clinical benefits of digital technologies are also aware of the financial investments required for their implementation. The association between O1 and TD4, as well as TD5, indicates that advanced clinical digital technologies are more frequently perceived in relation to economic barriers generated by high acquisition and integration costs. The links between O7 (preference for conventional methods) and TD3 (intraoral scanning/digital impression), as well as TD5 (occlusal analysis tools/software), are maintained in 2025, potentially reflecting difficulties in adapting to digital technologies that influence clinical and functional stages of dental treatment. The associations between O7 and M4 (productivity increase), as well as M8 (use as a marketing tool), suggest that respondents oriented toward conventional methods tend to perceive the benefits of digital technologies to a lesser extent in terms of practice efficiency and professional promotion. This may indicate a perceptual difference between digitally oriented users and those who prefer traditional clinical workflows.

The present study has certain methodological limitations that should be considered when interpreting the results. First, the study was conducted locally among dentists in Sibiu County, which limits the generalizability of the findings to the entire population of dentists in Romania, given potential regional differences in digital infrastructure, income levels, and access to professional training. Second, the voluntary nature of participation may introduce selection bias. The sample may overrepresent dentists who are more professionally active or more inclined to use digital technologies, which could influence the overall picture of technology adoption levels and attitudes toward digitalization. Furthermore, the comparison between the two analyzed periods (2022 and 2025) should be interpreted with caution, as the samples were independent and did not include the same participants, which may affect the comparative interpretation of the results. Also, because multiple comparisons were evaluated without statistical adjustment, findings should be interpreted as exploratory rather than confirmatory. Despite these limitations, the study provides a coherent and relevant perspective on current trends in digitalization within dentistry in Sibiu and may serve as a starting point for future research extended to a regional or national level.

## Conclusion

The study highlights a clear evolution of the digitalization process among dentists in Sibiu County, reflected in the increased use of basic digital technologies and the gradual adoption of advanced digital tools. Patient management software and intraoral scanning have become integrated components of the clinical workflow, confirming the transition from initial interest to effective implementation. At the same time, complex technologies such as CAD/CAM systems, 3D printing, and digital occlusal analysis, although still limited in adoption, benefit from growing interest and a positive perception of their clinical potential.

The results emphasize the importance of continuing education and professional training as key determinants in accelerating implementation, as well as the need for financial support to overcome the identified economic barriers. Moreover, the findings suggest a professional community with a generally positive attitude toward digital innovation and the adoption of new technologies.

In conclusion, dental practice digitalization in Sibiu County progressed between 2022 and 2025, with increased adoption of digital technologies, while further development may depend on addressing barriers related to training, implementation costs, and integration into clinical workflows.

## Data Availability

The data presented in this study are available upon reasonable request from the corresponding author.
